# Effect of *Polygonatum sibiricum* on biological toxicity of zinc oxide nanoparticles during respiratory exposure

**DOI:** 10.1039/d4ra03738c

**Published:** 2024-10-02

**Authors:** Jingjing Yao, Wanqing Yang, Liang Tang, Dicheng Yang, Yan Xu, Shenmin Zhu, Jun Zhu

**Affiliations:** a State Key Laboratory of Metal Matrix Composites, Shanghai Jiao Tong University Shanghai 200240 People's Republic of China yzjzhu@163.com smzhu@sjtu.edu.cn; b National Engineering Research Center for Nanotechnology Shanghai 200241 People's Republic of China; c Department of Orthopedic Surgery, Tongren Hospital, Shanghai Jiao Tong University School of Medicine 1111 XianXia Road Shanghai 200336 People's Republic of China

## Abstract

Although zinc oxide nanoparticles (ZnO NPs) with distinct physicochemical properties have attracted great attention, the application of ZnO NPs is still limited due to their potential biotoxicity. In this work, ZnO-*Polygonatum sibiricum* (PS) NPs are synthesized to overcome this challenge. The ZnO NPs stably combine with PS according to microstructural observation, particle size distribution, zeta potential results and Fourier transform infrared spectroscopy analysis. The cytotoxicity of ZnO NPs is alleviated by combining them with PS as a consequence of the diminished generation of reactive oxygen species and reinforced superoxide dismutase activity. Furthermore, the respiratory index and histopathologic results of mice exposed to NPs manifest that the pulmonary dysfunction caused by ZnO NPs is avoided in the ZnO-PS NPs group. This study provides the foundations for the amelioration and universal utilization of ZnO NPs and emphasizes the potential of ZnO-PS NPs in biomedical applications.

## Abstract 2

ZnO-PS NPs are synthesized to reduce the pulmonary toxicity of inhaled ZnO NPs through attaching finer ZnO NPs and endowing them with the desirable antioxidant activity of the composites.

## Graphical abstract

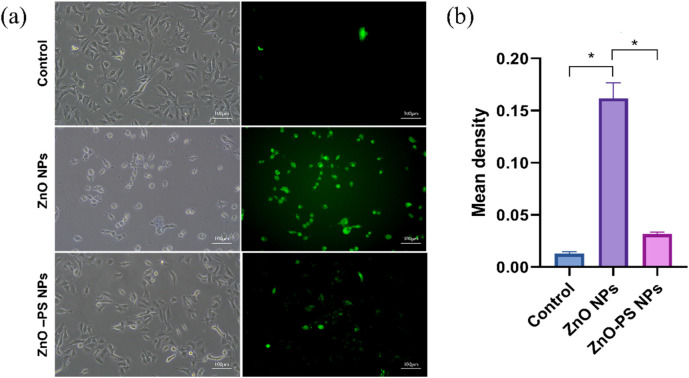

## Introduction

Zinc oxide nanoparticles (ZnO NPs) have recently become prevalent in sunscreens, biosensors, and antibacterial agents on account of their unique physicochemical characteristics.^1,2^ The toxicity potential of ZnO NPs has become a growing concern with the inevitable exposure to ZnO NPs in daily life.^3,4^ The underlying mechanism of toxicity has been reported to originate from the dissolution of ZnO NPs and the generation of reactive oxygen species (ROS), which results in redox system imbalance, lysosomal and mitochondrial damage, pro-inflammatory responses, cell death, and lung fibrosis.^5–8^ In particular, the pulmonary damage triggered by inhalation of ZnO NPs is shown to be concentration-independent since ZnO NPs can exert toxic effects regardless of being dissolved as Zn ions.^9^ Pulmonary surfactant inhibition owing to inhalation of ZnO NPs causes alveolar collapse and depression of the tidal volume through increasing the level of albumin proteins, associated with pulmonary surfactant inhibitors, and absorbing pulmonary surfactant components such as phospholipids, finally leading to pulmonary dysfunction, which has been found both in mice as well as in human aerosol challenge experiments.^10–12^ Therefore, the toxicity of ZnO NPs needs to be solved for their wide application.

In current studies, numerous methods have been tried to mitigate the toxicity of ZnO NPs by modifying the surface properties to inhibit the uptake of ZnO NPs into cells or their subsequent dissolution, such as organic functional groups or antibody grafting, combining with polymers or silica coating.^13–17^ Combining with polymers is one of the most popular ways to mediate the properties of ZnO NPs, which enables ZnO NPs to refrain from instability, aggregation and toxicity *via* properly entrapping or attaching NPs into the matrix without concentration limitation due to desirable biocompatibility and biodegradability.^18–20^ For instance, alginate was utilized to encapsulate ZnO NPs and gum acacia to accelerate wound healing, and the composites were verified to relieve inflammation in rabbits without scar formation.^21^ Reddy *et al.* introduced chitosan-l-ornithine to optimize ZnO NPs, and found that the antibacterial activity of ZnO NPs could be maintained while improving cell viability.^22^ However, the polymers are generally designed to participate in controlling the release of ZnO NPs or combining functional materials onto them. It is necessary to explore a polymer combined with ZnO NPs that can not only act as a carrier for NPs but also endow the composites with multifunctional properties.

*Polygonatum sibiricum* (PS), a traditional Chinese medicine containing phenolics and flavonoids, has drawn increasing attention as a new therapeutic material originating from its extraordinary antioxidant properties.^23–29^ Additionally, PS has been applied to functionalize metallic NPs in some studies in the literature. In terms of the slightly lower toxicity of SeNPs, Chen *et al.* utilized PS to modify SeNPs and found that the free radical scavenging ability of PSP-SeNPs for the H_2_O_2_-induced oxidative damage of cells was reinforced, presenting desirable oxidation resistance.^30^ In addition, PSP@AgNPs were synthesized as antibacterial agents against wound infections. The PSP@AgNPs with a regular and homogeneous microstructure displayed increasing antibacterial activity through inhibiting the relative enzyme activity and faster wound healing.^31^ In this study, ZnO-PS NPs have been prepared through combining PS with ZnO NPs to obtain a high-performance composite with low toxicity and desirable antioxidant behaviour, and we have investigated the effect of combination with PS on the cytotoxicity of the ZnO NPs group and the underlying mechanism *in vitro*. The toxicity of ZnO-PS NPs on the pulmonary system was also evaluated through respiratory exposure of mice to aerosolized NPs.

## Experimental

### Materials

PS was purchased from Shanxi Hongda Phytochemistry Co., Ltd (Shanxi, China). Commercial grade ZnO NPs were supplied by Sigma-Aldrich (USA), and dextrin was acquired from Sinopharm Chemical Reagent Co., Ltd (Shanghai, China).

### Preparation of ZnO-PS NPs

Dried PS after nine repeats of steaming and sun-drying was milled for 5 min with dextrin as dispersant at a ratio of 10 to 1; then the mixture was subjected to five cycles of high-pressure homogenization with a working pressure of 1600 bar. A dispersion of ZnO NPs was obtained by wet ball milling 10 times using zirconia beads with a diameter of 0.05 mm, and each ball was milled for 4 hours. The ZnO NP powders were mixed with various PS concentrations and stirred for 72 hours, followed by sonication for 30 minutes, and the PS and ZnO NP concentrations of ZnO-PS NPs with various ratios are listed in Table 1.

**Table 1:** Composition of materials in this study

	ZnO NPs	PS	ZnO-0.2PS NPs	ZnO-PS NPs	ZnO-5PS NPs	ZnO-10PS NPs
ZnO NPs concentration (μg mL^−1^)	5	5	5	5	5	5
PS concentration (μg mL^−1^)	—	—	1	5	25	50
Ratio of PS/ZnO content	—	—	0.2	1	5	10

### Characterization

Scanning electron microscopy (SEM; S-4800, Hitachi, Japan) and transmission electron microscopy (TEM; JEM-2100F, JEOL, Japan) were utilized to analyse the morphology of the materials. Dynamic light scattering (DLS) analysis was used to determine the zeta potential and particle size distribution with a Zetasizer Nano ZS (Malvern, UK). The Fourier transform infrared (FTIR) spectra were acquired with a Nicolet 6700 spectrophotometer (Thermo, USA).

### Cytotoxicity

A Cell Counting Kit-8 (CCK-8) assay (Yeasen, Shanghai, China) was employed to assess the impact of ZnO-PS NPs on the viability of human alveolar epithelial cells (HPAEpiC). 100 μL of cell suspension with a concentration of 5 × 10^4^ CFU mL^−1^ was seeded and treated with ZnO-PS NPs dispersed in serum-free medium for 24 h. Then, the optical density (OD) at 450 nm was measured with a microplate reader (Synergy LX, Bio-TEK, USA) after 3 h of incubation with 10 μL of CCK-8 detection reagent.

### Determination of intracellular ROS and SOD enzyme activity

A reactive oxygen species assay kit (Yeasen, Shanghai, China) was applied to explore ROS levels. A fluorescence microscope (M700E, NIKON, Japan) and ImageJ software were utilized to capture and quantify the fluorescence intensity of DCFH-DA, respectively. The superoxide dismutase (SOD) activity was determined using a Total Superoxide Dismutase Assay Kit following the manufacturer's instructions (S0101, Beyotime, Shanghai, China).

### Measurement of mitochondrial membrane potential

The mitochondrial membrane potential was detected using a JC-1 Mitochondrial Membrane Potential kit (Yeasen, Shanghai, China). After treatment with ZnO NPs and ZnO-PS NPs, HPAEpiC cells were incubated with JC-1 buffer for 20 min. The fluorescence intensities were recorded with a fluorescence microscope (M700E, NIKON, Japan) and quantitated with ImageJ software.

### Respiratory exposure of animals

BALB/c mice (weight 20 ± 2 g, 6–8 weeks) were used for the respiratory exposure experiment and all animal procedures were performed in accordance with the Guidelines for Care and Use of Laboratory Animals of Shanghai Jiao Tong University and experiments were approved by the Animal Ethics Committee of IACUCs. The mice were housed in individual cages and randomly assigned to three groups: ZnO NPs group, ZnO-PS NPs group, and a normal control group. Each group was subjected to exposure every two days and each exposure was continued for 3 hours. Before exposure, all nanoparticles were aerosolized using an NSF-6A liquid aerosol generator (Tawang Technology, Shanghai).

### Measurement of respiratory indicators

The respiratory indicators were obtained by using a whole-body plethysmography (WBP) system (Tawang Technology, Shanghai). Before each collection, mice were placed in the collection bin for 30 minutes to stabilize their breathing for valid data.

### Histopathology

The tissues were fixed with 10% formaldehyde. The tissue samples were then cleaned and dehydrated in alcohol. The dehydrated samples were embedded in parafilm to produce fine sections followed by staining with hematoxylin and eosin (H&E).

### Statistical analysis

Each experiment was conducted with no less than three duplicates, and the mean ± standard deviation was used to display the data. A Student's *t*-test (two-tailed, equal variances) was utilized to assess statistical differences among groups with GraphPad software. Statistical significance was established at a significance level of *p* < 0.05.

## Results and discussion

**Fig. 1:**
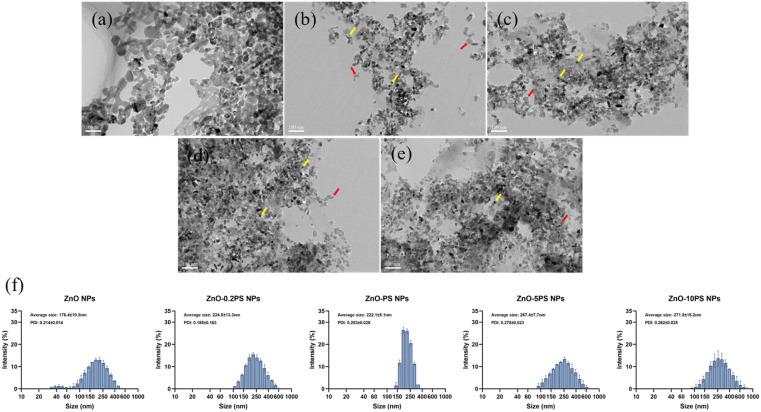
Morphological images of (a) ZnO NPs; (b) ZnO-0.2 PS NPs; (c) ZnO-PS NPs; (d) ZnO-5PS NPs; and (e) ZnO-10PS NPs. (f) DLS particle size distribution of NPs.

The morphologies of ZnO NPs and ZnO-PS NPs with various PS concentrations were observed by TEM. The spherical-like ZnO NPs (yellow arrows) and irregular PS (marked with red arrows) are apparent in Fig. 1(a–e). It is found that the nanoparticles gather together in purely ZnO NPs, which stably attach to PS in ZnO-PS NPs with various PS concentration groups. The large agglomerates readily form with increasing PS concentration since the excessive PS chains combine with ZnO NPs.^30^ The particle size distribution of NPs obtained by DLS is displayed in Fig. 1(f) and the average particle size of the NPs is listed in Table 2. There exist NPs with sizes of around 40 nm and larger than 100 nm in the ZnO NPs group resulting from aggregates of NPs, while NPs with sizes below about 100 nm disappear in the ZnO-PS NPs groups. The average particle size of ZnO-PS NPs with various PS concentrations increases with increasing PS concentration, which are 224.5 ± 13.3 nm, 222.1 ± 5.1 nm, 267.4 ± 7.7 nm and 271.5 ± 15.2 nm for the ZnO-0.2PS NPs, ZnO-PS NPs group, ZnO-5PS NPs and ZnO-10PS NPs group, respectively. This tendency is consistent with the TEM results, which can mainly be attributed to the combination of ZnO NPs and PS. Furthermore, the zeta potential of the NPs was detected to analyze the stability of the NPs, as presented in Table 2, which were about 2.4 ± 1.3 mV, −21.4 ± 3.5 mV, −20.8 ± 0.9 mV, −16.9 ± 1.9 mV and −18.0 ± 1.2 mV for the ZnO NPs, ZnO-0.2PS NPs, ZnO-PS NPs, ZnO-5PS NPs and ZnO-10PS NPs groups, respectively. It can be seen that the zeta potential of ZnO-PS NPs is more negative than that of ZnO NPs, implying the stability of the combination.^32^

**Table 2:** The average particle size and zeta potential of ZnO NPs, PS and ZnO-PS NPs with various PS concentrations in water medium

	ZnO NPs	ZnO-0.2PS NPs	ZnO-PS NPs	ZnO-5PS NPs	ZnO-10 PS NPs
PS/ZnO	—	0.2	1	5	10
Average size (nm)	176.4 ± 10.3	224.5 ± 13.3	222.1 ± 5.1	267.4 ± 7.7	271.5 ± 15.2
Zeta potential (mV)	+2.4 ± 1.3	−20.8 ± 0.6	−22.5 ± 0.6	−26.2 ± 0.3	−30.8 ± 0.5

**Fig. 2:**
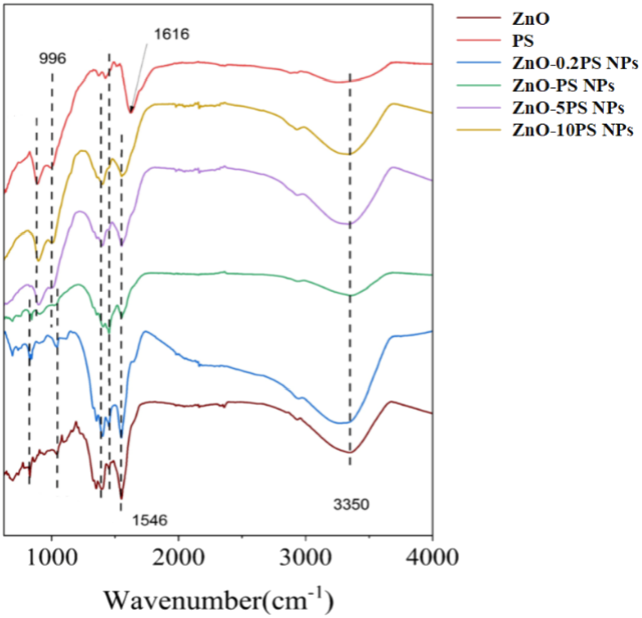
FT-IR spectra of various NPs.

The FT-IR spectra of ZnO NPs, PS and ZnO-PS NPs with various PS concentrations are illuminated in Fig. 2. In the PS spectrum, the distinctive peaks at 3350 cm^−1^, 1616 cm^−1^, and 996 cm^−1^ can be seen, caused by the stretching vibration of the O–H, the C=C and the C–O–C ether bond on the pyranoside, respectively. Moreover, ZnO-PS NPs with various PS concentrations exhibit the characteristic peaks of PS. It is noteworthy that the peak of C=C stretching vibration in ZnO-PS NPs is shifted to 1546 cm^−1^, which is mainly ascribed to the intermolecular and intramolecular hydrogen bonds between ZnO and PS functional groups.^27^ Hence, the FTIR spectra also verify the combination of PS and ZnO NPs.

**Fig. 3:**
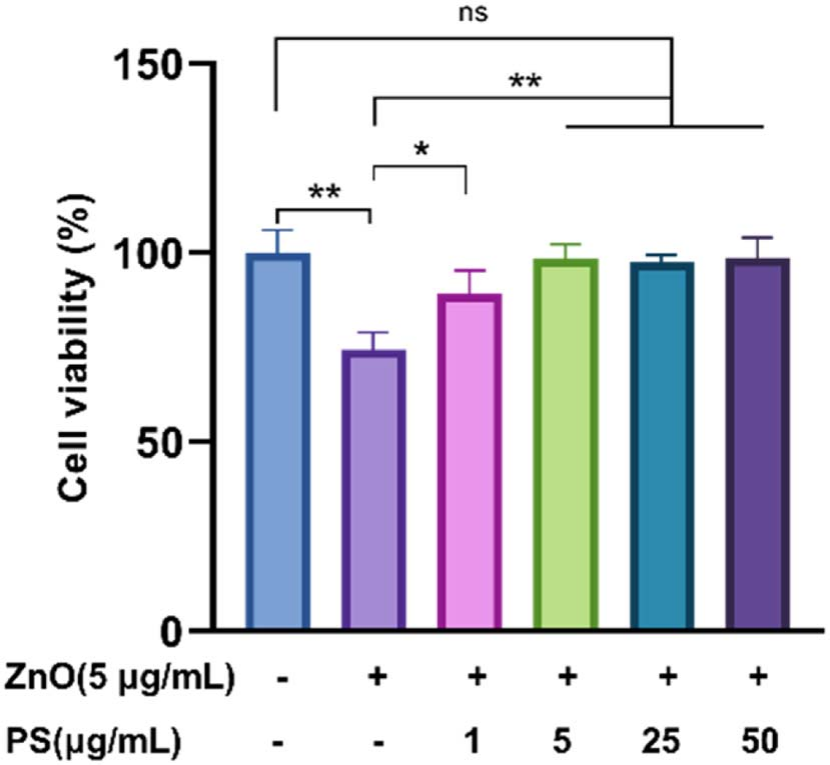
Cell viability assessment of HPAEpiC cells treated by various NPs. Data from three separate experiments are expressed as mean (SD). *P < 0.05. **P < 0.01. “ns” indicates no significance.

Then the biological toxicity of ZnO-PS NPs was studied. The cell viability of HPAEpiC cells was evaluated after coincubation with ZnO-PS NPs for 24 h. The results in Fig. 3 depict that the cell viability of the ZnO NPs group drops to 74.5%, showing the nonnegligible cytotoxicity of ZnO NPs, which is similar to the value in previous literature.^12^ It is worth noting that the cell survival rates of the ZnO-0.2PS NPs, ZnO-PS NPs, ZnO-5PS NPs and ZnO-10PS NPs groups are 89.2%, 98.3%, 97.7% and 98.6%, respectively, which implies an obvious improvement in cell survival with increasing PS concentration. Consequently, PS plays a positive role in reducing the cytotoxicity of ZnO NPs.

**Fig. 4:**
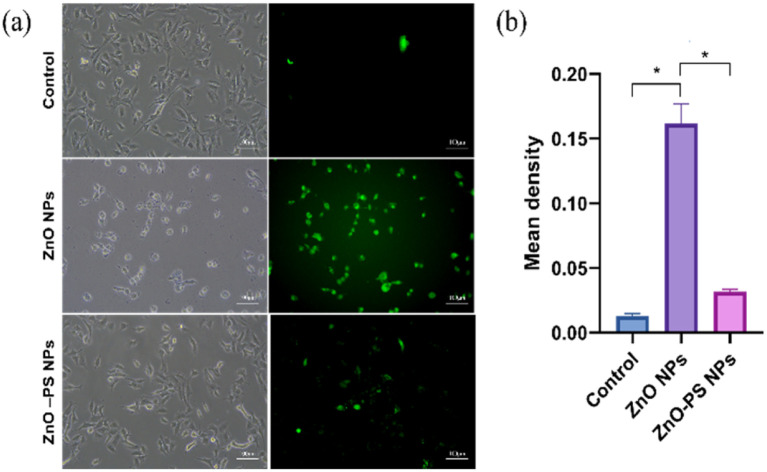
Morphology of HPAEpiC cells in control group, ZnO NPs group and ZnO-PS NPs group and corresponding intracellular ROS detection images (a); quantification of fluorescent intensity (b). Data from three separate experiments are expressed as mean (SD). *p < 0.05.

**Fig. 5:**
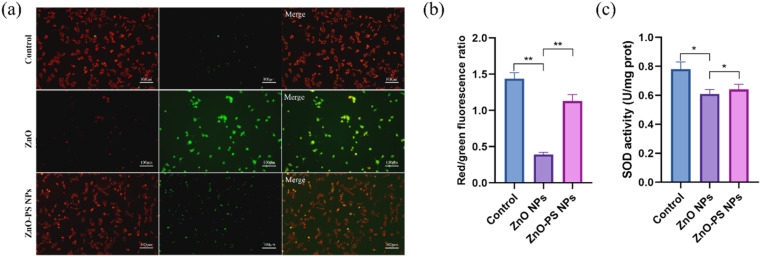
Fluorescence images of JC-1 (a); quantitative assessment of red to green fluorescence ratio (b); SOD enzyme activity (c). Data from three separate experiments are expressed as mean (SD). *P < 0.01 *versus* the control group; *P < 0.05 *versus* the control; ##*P* < 0.01 *versus* ZnO NPs group; *P < 0.05; **P < 0.01.

Based on the above results, the optimal ZnO-PS NPs group (PS concentration of 5 μg mL^−1^) with desirable biocompatibility was used to further reveal the mechanism underlying the reduction of cytotoxicity of the ZnO-PS NPs group, and the ROS levels, mitochondrial membrane potential and SOD activities of HPAEpiC cells treated with ZnO-PS NPs were evaluated. The intracellular ROS levels of cells treated with ZnO NPs and ZnO-PS NPs are quantified in Fig. 4. The ZnO NPs group exhibits a stronger fluorescence intensity (0.013 ± 0.002) than that of the control group (0.162 ± 0.015), implying that exposure to ZnO NPs could induce ROS production in HPAEpiC cells. This phenomenon was also discussed in our previous work.^33^ Whereas the ROS level of the ZnO-PS NPs group (0.032 ± 0.003) is much lower than that in ZnO NPs group, which is comparable to that in the control group. In other words, PS is conducive to inhibiting the production of ROS caused by ZnO NPs. Furthermore, the mitochondrial membrane potential was detected through JC-1 staining, which embodies the damage to mitochondria. In normal mitochondria, JC-1 aggregates in the matrix and exists as a polymer emitting red fluorescence while it emits green fluorescence as a monomer in the damaged mitochondria. Fig. 5(a) and (b) show the fluorescence images of JC-1 and the statistical results of the red to green fluorescence ratio. The green fluorescence is dominant in the ZnO NPs group, which is weaker in the ZnO-PS NPs group. And the quantification of the red to green fluorescence ratio also shows that the degree of mitochondrial damage is diminished by combining PS with ZnO NPs. In addition, it is found that the SOD activity improves in the ZnO-PS NPs group by contrast with the ZnO NPs group, as revealed in Fig. 5(c).

**Fig. 6:**
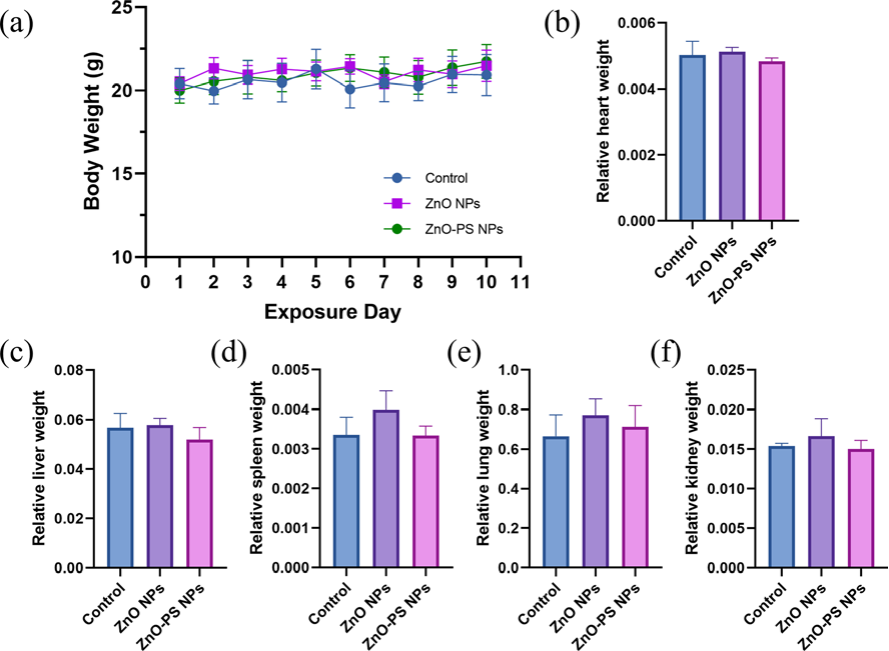
Mice weight change during exposure (a); relative heart weight (b); relative liver weight (c); relative spleen weight (d); relative lung weight (e); relative kidney weight (f) of various groups after exposure. Data from three separate experiments are expressed as mean (SD).

**Fig. 7:**
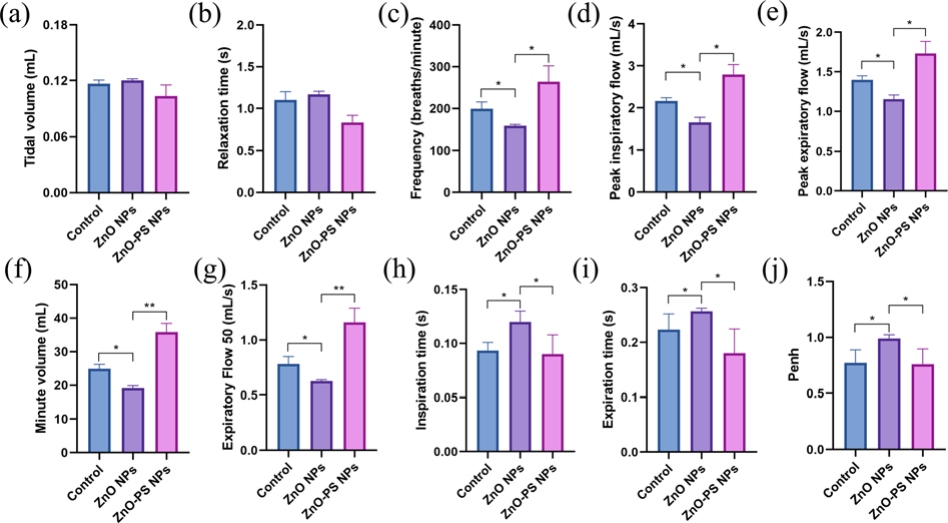
Respiratory indexes in mice after exposure. (a) Tidal volume; (b) relaxation time; (c) frequency; (d) peak inspiration flow; (e) peak expiratory flow; (f) minute volume; (g) expiratory flow 50; (h) inspiration time; (i) expiratory time; (j) Penh. Data from three separate experiments are expressed as mean (SD). *P < 0.05; **P < 0.01.

The effect of PS on the toxicity of ZnO NPs *in vivo* was investigated through respiratory exposure. Fig. 6 exhibits the change in body weight and the relative organ weight of mice after exposure. There is no discernible variation in body weight among the different groups during the exposure period, as shown in Fig. 6(a). Furthermore, the results in Fig. 6(b–f) indicate that exposure has little effect on the relative organ weights of various groups, resulting primarily from pulmonary tolerance to relatively low dose exposure to nanoparticles. However, dyspnea and hypoxia in mice in the ZnO NPs group are observed during the exposure period. Thus, the expiratory and inspiratory-related indexes were monitored to further assess the pulmonary function of the mice after exposure. Tidal volume (*T*_v_), relaxation time (RT, *i.e.*, the time necessary to expire 65% of *T*_v_), frequency (*f*), peak inspiration flow (PIF), peak expiratory flow (PEF), minute volume (*M*_v_), expiratory flow 50 (EF50), inspiration time (*T*_i_), expiratory time (*T*_e_) and Penh (an index of airflow limitation or bronchoconstriction calculated using the formula: Penh = PEF/PIF × (*T*_e_ – *R*_T_)/*R*_T_) are obtained through a whole-body plethysmography (WBP) system. According to the results in Fig. 7(a and b), there is no pronounced difference in tidal volume among all groups and the slight change in relaxation time among the control, ZnO NPs and ZnO-PS NPs groups, which are 1.10 ± 0.11 s, 1.17 ± 0.04 s and 0.83 ± 0.08 s, respectively, can be ignored as discussed in previous studies.^34^ However, the breath frequency of the ZnO NPs group mice is 0.8 times lower than that of the control group, accompanied by the phenomena that the peak inspiratory flow, peak expiratory flow, minute volume and EF50 of mice treated with ZnO NPs decrease remarkably, whereas the inspiration time and expiration time increase compared to those of the control group, as exhibited in Fig. 7(c–i). Moreover, the Penh value of the ZnO NPs group mice rises to 0.99, as revealed in Fig. 7(j). It is worth noting that all these indexes are rectified in the PS-ZnO NPs group.

**Fig. 8:**
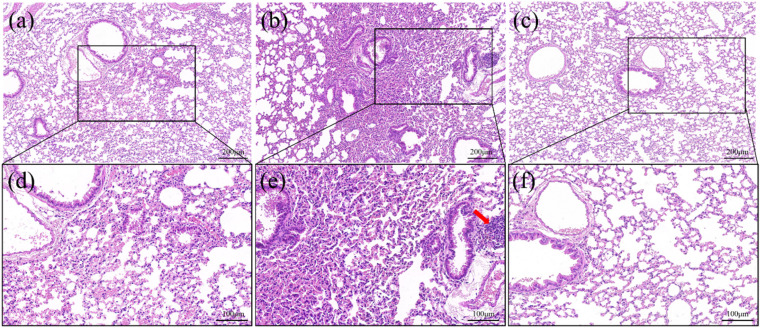
H&E staining images of lung tissue of mice treated by control (a and d), ZnO NPs (b and e) and ZnO-PS NPs group (c and f).

The H&E staining of lung tissue sections of mice of the various groups are shown in Fig. 8. There are striking differences in lung tissue among the groups. This discloses that the lung structure of the control group maintains normal cell morphology without any discernible apoptosis or apparent pathogenic alterations, as shown in Fig. 8(a). Nevertheless, inflammatory infiltration (red arrow in Fig. 8(e)) and a narrowed alveolar septum are observed in the ZnO NPs group (Fig. 8(b)). Interestingly, pathogenic alterations are avoided in the ZnO-PS NPs group (Fig. 8(c)). In brief, PS is favourable for decreasing the pulmonary toxicity of inhaled ZnO NPs.

The toxicity of ZnO NPs is an intractable challenge for their wide use in biomedical applications. The cell survival rate of ZnO NP treated cells is significantly reduced, as displayed in Fig. 3, and the underlying mechanisms are generally thought to be due to multiple factors, including membrane disruption and dysregulation of the redox system.^5,35^ The generation of ROS as a result of a rise in Zn ions driven by ZnO NP dissolution leads to redox system dysregulation, mitochondrial dysfunction and cell apoptosis.^7,36^ Thus, the dissolution of ZnO NPs is assumed to be a major trigger of cytotoxicity. In addition, previous studies suggested that the pulmonary damage caused by the inhalation of ZnO NPs through increasing the level of albumin proteins and absorbing pulmonary surfactant components such as phospholipids is concentration-independent since ZnO NPs can exert toxic effects regardless of being dissolved as Zn^2+^ ions.^9^ Here, we synthesized ZnO-PS NPs to reduce the toxicity of ZnO NPs, where the ZnO NPs stably combine with PS according to the TEM observations, particle sizes, zeta potential and FTIR spectral analysis, as illuminated in Fig. 1 and 2. All the above results prove that ZnO-PS NPs possess lower toxicity than ZnO NPs. We tentatively put forward that the combination hinders aggregation of ZnO NPs and the dissolution of dispersed ZnO NPs, thus improving the cell viability of the ZnO-PS NPs group through maintaining the redox system balance of the cells (Fig. 4 and 5). On the other hand, the toxicity of ZnO NPs is also rectified in the ZnO-PS NPs group by strengthening the SOD activity, as shown in Fig. 5(c), which is probably a consequence of the antioxidant ability of PS, so it has been confirmed that PS can regulate the levels of ROS since it can react with free radicals and terminate radical chain reactions.^25,37^ Last but not least, the results of the *in vivo* experiment in Fig. 7 and 8 reveal depression of the inflammatory cells and the narrowed alveolar septum in the ZnO NPs group resulting from the shear stress of fine ZnO NPs and exposure to Zn ions, which is avoided in the ZnO-PS NPs group due to the combination with PS.

## Conclusions

This study provides a facile approach to reducing the toxicity of ZnO NPs through synthesizing ZnO-PS NPs. The results display the dispersed distribution of ZnO-PS NPs with a stable structure. Most importantly, the cytotoxicity of ZnO NPs is reduced by the combination of PS and ZnO NPs contributing to the desirable antioxidant ability of the composite. Moreover, the respiratory exposure experiments demonstrate that the ZnO-PS NPs group diminishes the passive effect of ZnO NPs on lung tissue. Our findings provide foundations for the amelioration of ZnO NPs for universal application and emphasize the potential of ZnO-PS NPs in biomedical applications. However, the specific antioxidant kinetics of ZnO-PS NPs remain to be further explored.

## Data availability

The authors confirm that the data supporting the findings of this study are available within the article.

## Author contributions

Jingjing Yao: investigation, methodology, data curation, writing – original draft. Wanqing Yang: investigation, methodology. Liang Tang, Dicheng Yang: methodology, supervision. Yan Xu: writing – review & editing, supervision. Shenmin Zhu, Jun Zhu: conceptualization, writing – review & editing, funding acquisition.

## Conflicts of interest

The authors declare that they have no known competing financial interests or personal relationships that could have appeared to influence the work reported in this paper.
